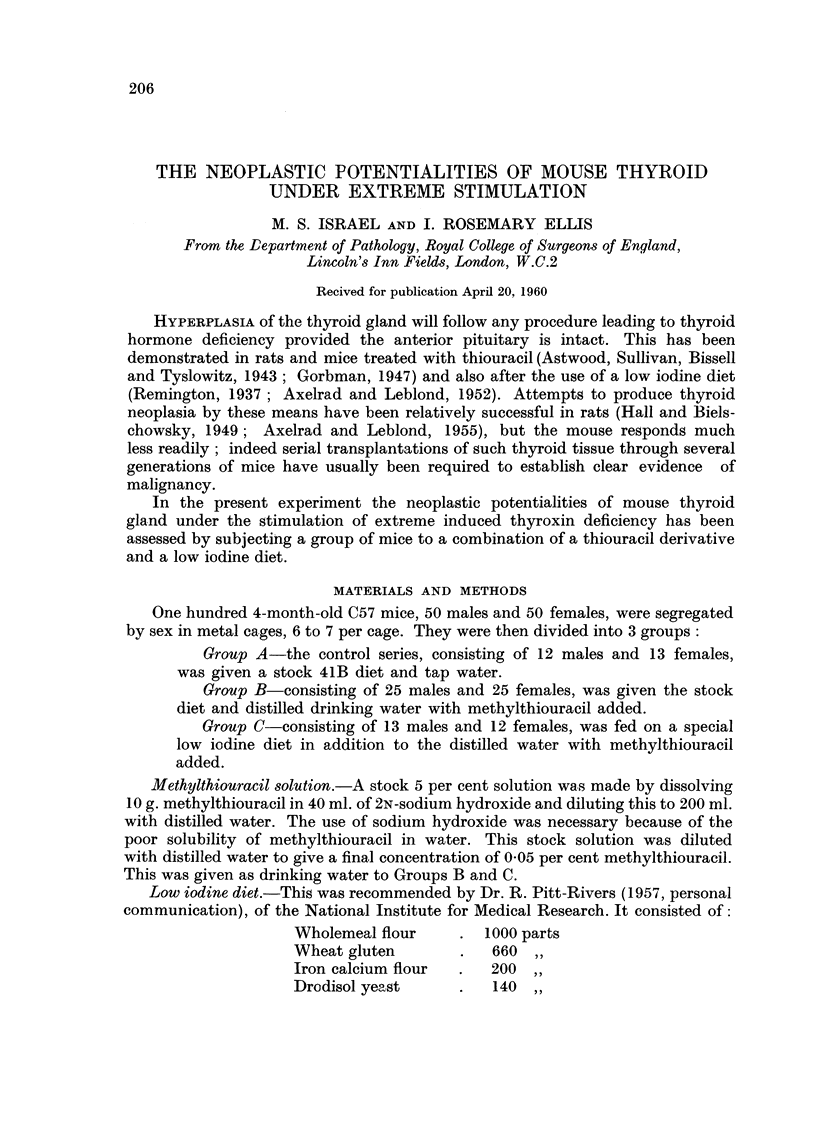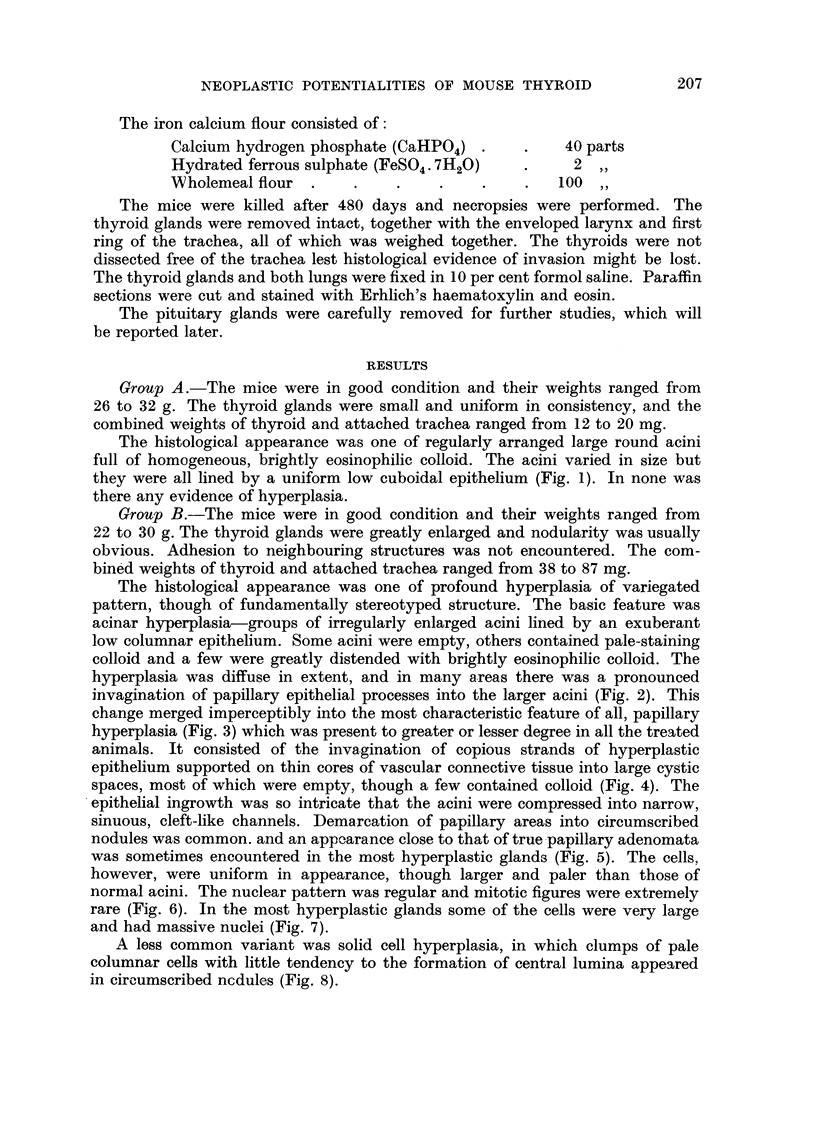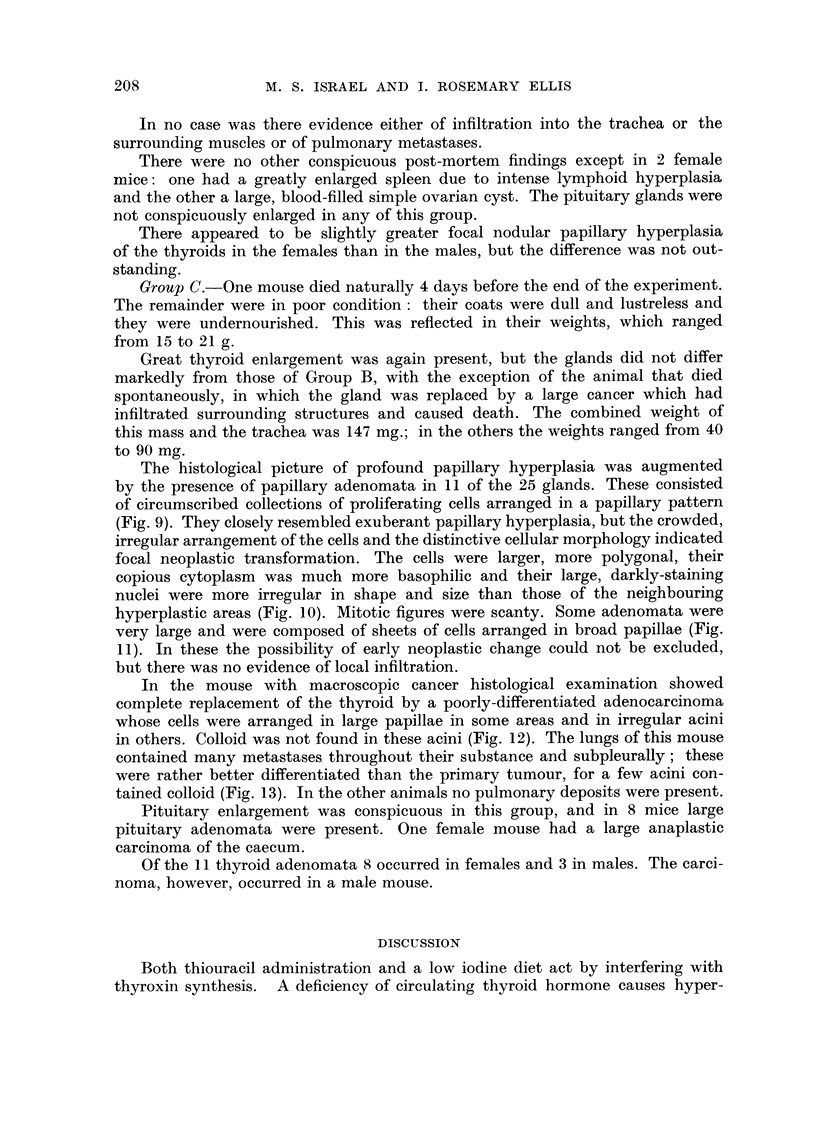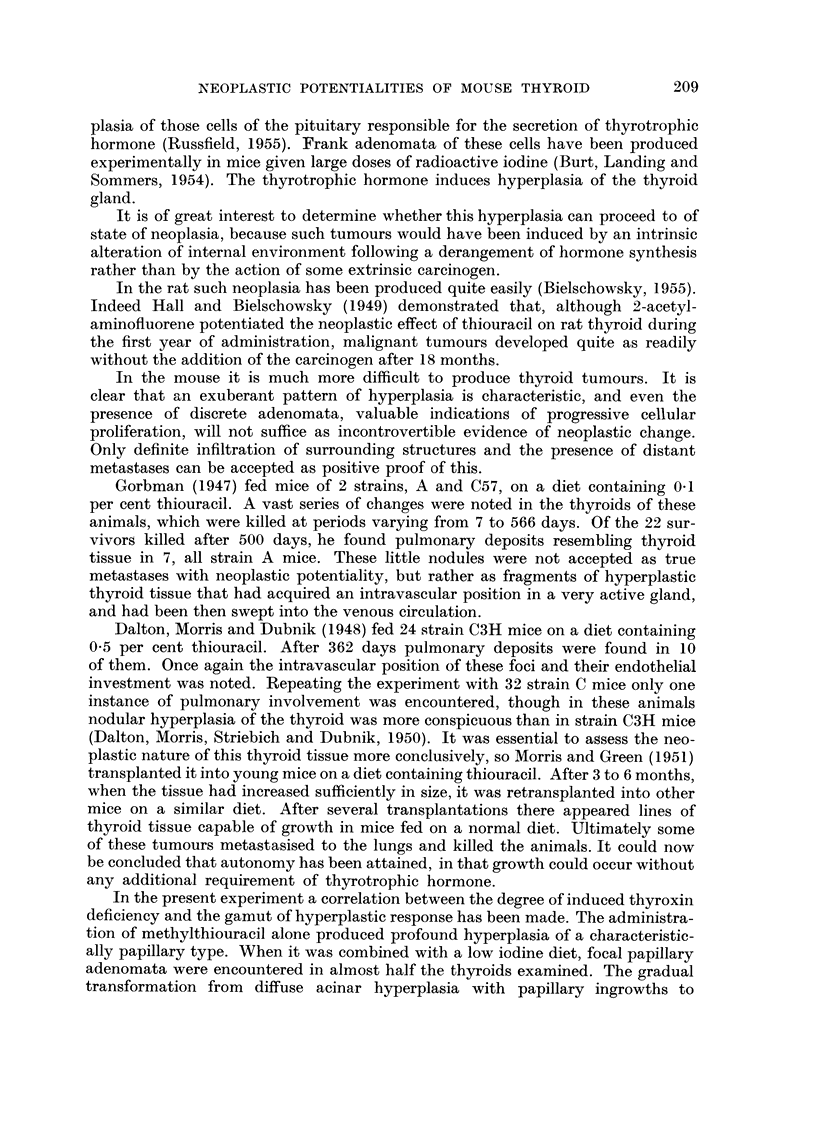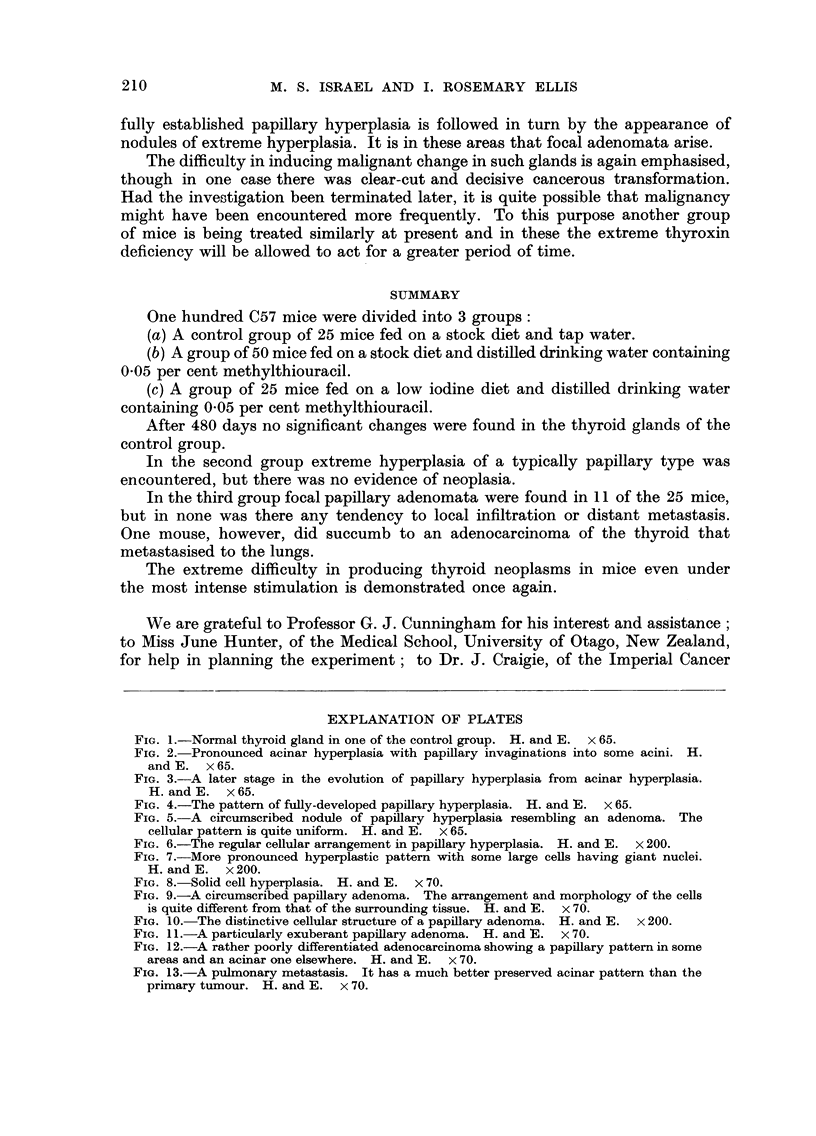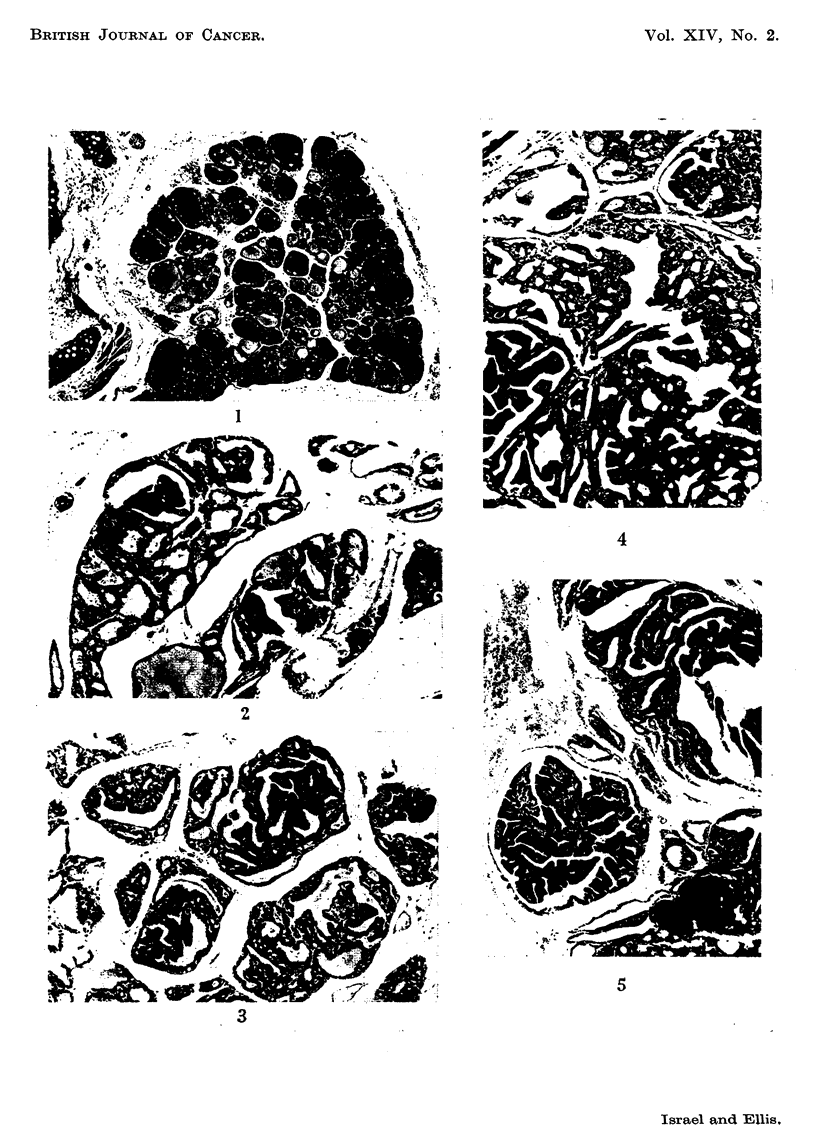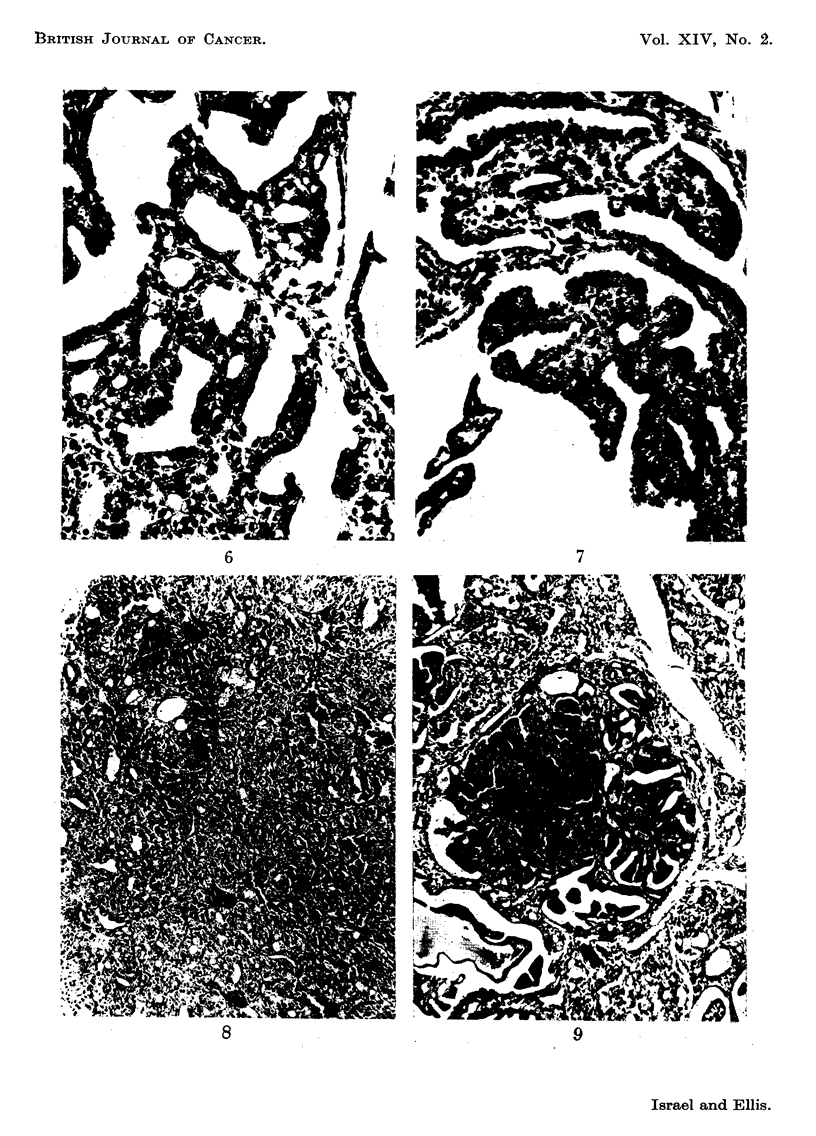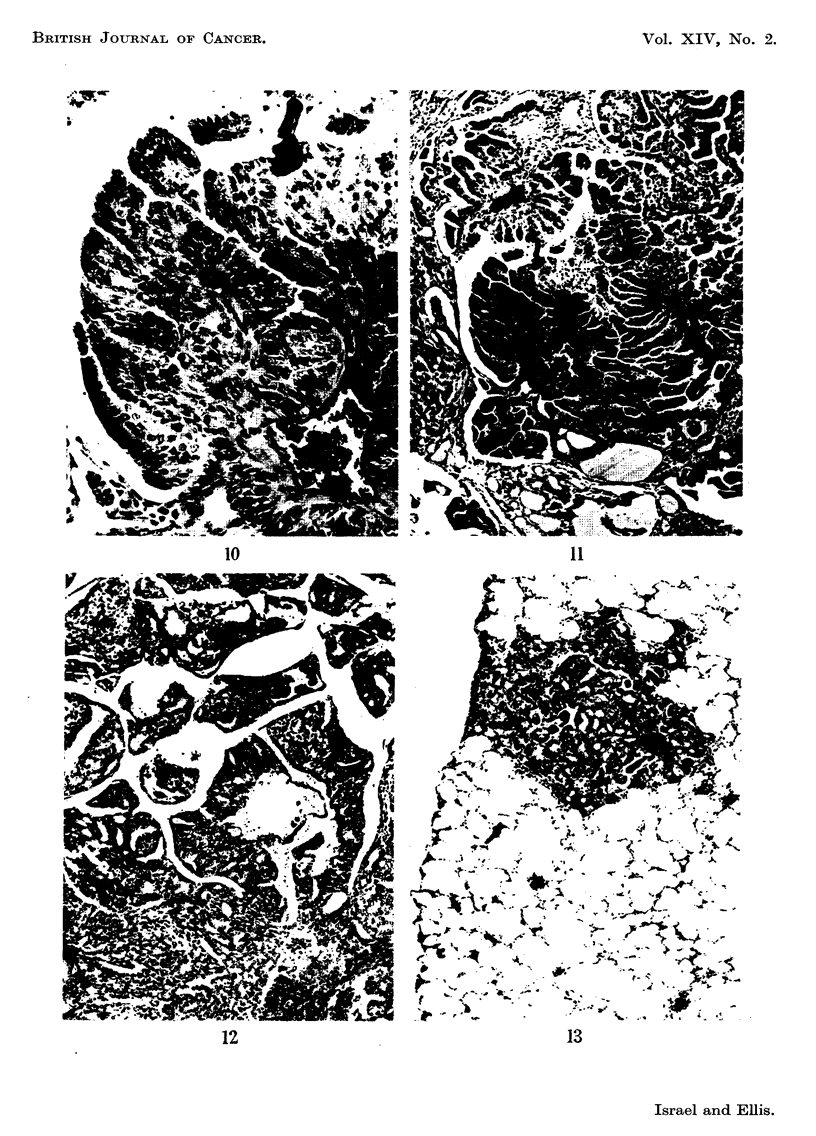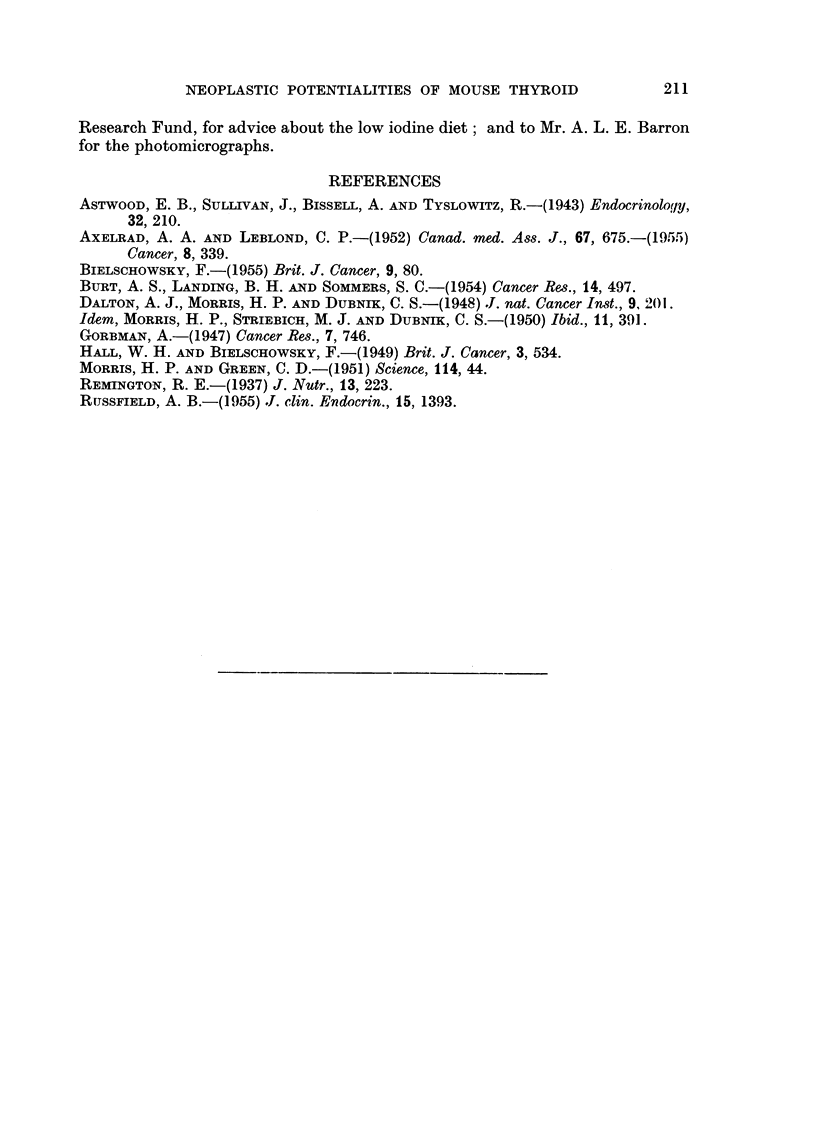# The Neoplastic Potentialities of Mouse Thyroid under Extreme Stimulation

**DOI:** 10.1038/bjc.1960.25

**Published:** 1960-06

**Authors:** M. S. Israel, I. Rosemary Ellis

## Abstract

**Images:**


### THE NEOPLASTIC POTENTIALITIES OF MOUSE THYROID UNDER EXTREME STIMULATION

M. S. ISRAEL AND I. ROSEMARY ELLIS

*From the Department of Pathology, Royal College of Surgeons of England, Lincoln's Inn Fields, London, W.C.2*

Recived for publication April 20, 1960

HYPERPLASIA of the thyroid gland will follow any procedure leading to thyroid hormone deficiency provided the anterior pituitary is intact. This has been demonstrated in rats and mice treated with thiouracil (Astwood, Sullivan, Bissell and Tyslowitz, 1943 ; Gorbman, 1947) and also after the use of a low iodine diet (Remington, 1937 ; Axelrad and Leblond, 1952). Attempts to produce thyroid neoplasia by these means have been relatively successful in rats (Hall and Bielschowsky, 1949 ; Axelrad and Leblond, 1955), but the mouse responds much less readily ; indeed serial transplantations of such thyroid tissue through several generations of mice have usually been required to establish clear evidence of malignancy.

In the present experiment the neoplastic potentialities of mouse thyroid gland under the stimulation of extreme induced thyroxin deficiency has been assessed by subjecting a group of mice to a combination of a thiouracil derivative and a low iodine diet.

### MATERIALS AND METHODS

One hundred 4-month-old C57 mice, 50 males and 50 females, were segregated by sex in metal cages, 6 to 7 per cage. They were then divided into 3 groups :

*Group A*—the control series, consisting of 12 males and 13 females, was given a stock 41B diet and tap water.

*Group B*—consisting of 25 males and 25 females, was given the stock diet and distilled drinking water with methylthiouracil added.

*Group C*—consisting of 13 males and 12 females, was fed on a special low iodine diet in addition to the distilled water with methylthiouracil added.

*Methylthiouracil solution.*—A stock 5 per cent solution was made by dissolving 10 g. methylthiouracil in 40 ml. of 2N-sodium hydroxide and diluting this to 200 ml. with distilled water. The use of sodium hydroxide was necessary because of the poor solubility of methylthiouracil in water. This stock solution was diluted with distilled water to give a final concentration of 0·05 per cent methylthiouracil. This was given as drinking water to Groups B and C.

*Low iodine diet.*—This was recommended by Dr. R. Pitt-Rivers (1957, personal communication), of the National Institute for Medical Research. It consisted of :

| | | |
|---|---|---|
| Wholemeal flour | . | 1000 parts |
| Wheat gluten | . | 660 ,, |
| Iron calcium flour | . | 200 ,, |
| Drodisol yeast | . | 140 ,, |

The iron calcium flour consisted of:

| | |
|---|---|
| Calcium hydrogen phosphate ($CaHPO_4$) . . | 40 parts |
| Hydrated ferrous sulphate ($FeSO_4.7H_2O$) . | 2 ,, |
| Wholemeal flour . . . . . . . | 100 ,, |

The mice were killed after 480 days and necropsies were performed. The thyroid glands were removed intact, together with the enveloped larynx and first ring of the trachea, all of which was weighed together. The thyroids were not dissected free of the trachea lest histological evidence of invasion might be lost. The thyroid glands and both lungs were fixed in 10 per cent formol saline. Paraffin sections were cut and stained with Erhlich's haematoxylin and eosin.

The pituitary glands were carefully removed for further studies, which will be reported later.

### RESULTS

*Group A.*—The mice were in good condition and their weights ranged from 26 to 32 g. The thyroid glands were small and uniform in consistency, and the combined weights of thyroid and attached trachea ranged from 12 to 20 mg.

The histological appearance was one of regularly arranged large round acini full of homogeneous, brightly eosinophilic colloid. The acini varied in size but they were all lined by a uniform low cuboidal epithelium (Fig. 1). In none was there any evidence of hyperplasia.

*Group B.*—The mice were in good condition and their weights ranged from 22 to 30 g. The thyroid glands were greatly enlarged and nodularity was usually obvious. Adhesion to neighbouring structures was not encountered. The combined weights of thyroid and attached trachea ranged from 38 to 87 mg.

The histological appearance was one of profound hyperplasia of variegated pattern, though of fundamentally stereotyped structure. The basic feature was acinar hyperplasia—groups of irregularly enlarged acini lined by an exuberant low columnar epithelium. Some acini were empty, others contained pale-staining colloid and a few were greatly distended with brightly eosinophilic colloid. The hyperplasia was diffuse in extent, and in many areas there was a pronounced invagination of papillary epithelial processes into the larger acini (Fig. 2). This change merged imperceptibly into the most characteristic feature of all, papillary hyperplasia (Fig. 3) which was present to greater or lesser degree in all the treated animals. It consisted of the invagination of copious strands of hyperplastic epithelium supported on thin cores of vascular connective tissue into large cystic spaces, most of which were empty, though a few contained colloid (Fig. 4). The epithelial ingrowth was so intricate that the acini were compressed into narrow, sinuous, cleft-like channels. Demarcation of papillary areas into circumscribed nodules was common. and an appcarance close to that of true papillary adenomata was sometimes encountered in the most hyperplastic glands (Fig. 5). The cells, however, were uniform in appearance, though larger and paler than those of normal acini. The nuclear pattern was regular and mitotic figures were extremely rare (Fig. 6). In the most hyperplastic glands some of the cells were very large and had massive nuclei (Fig. 7).

A less common variant was solid cell hyperplasia, in which clumps of pale columnar cells with little tendency to the formation of central lumina appeared in circumscribed ncdules (Fig. 8).

In no case was there evidence either of infiltration into the trachea or the surrounding muscles or of pulmonary metastases.

There were no other conspicuous post-mortem findings except in 2 female mice: one had a greatly enlarged spleen due to intense lymphoid hyperplasia and the other a large, blood-filled simple ovarian cyst. The pituitary glands were not conspicuously enlarged in any of this group.

There appeared to be slightly greater focal nodular papillary hyperplasia of the thyroids in the females than in the males, but the difference was not outstanding.

*Group C*.—One mouse died naturally 4 days before the end of the experiment. The remainder were in poor condition : their coats were dull and lustreless and they were undernourished. This was reflected in their weights, which ranged from 15 to 21 g.

Great thyroid enlargement was again present, but the glands did not differ markedly from those of Group B, with the exception of the animal that died spontaneously, in which the gland was replaced by a large cancer which had infiltrated surrounding structures and caused death. The combined weight of this mass and the trachea was 147 mg.; in the others the weights ranged from 40 to 90 mg.

The histological picture of profound papillary hyperplasia was augmented by the presence of papillary adenomata in 11 of the 25 glands. These consisted of circumscribed collections of proliferating cells arranged in a papillary pattern (Fig. 9). They closely resembled exuberant papillary hyperplasia, but the crowded, irregular arrangement of the cells and the distinctive cellular morphology indicated focal neoplastic transformation. The cells were larger, more polygonal, their copious cytoplasm was much more basophilic and their large, darkly-staining nuclei were more irregular in shape and size than those of the neighbouring hyperplastic areas (Fig. 10). Mitotic figures were scanty. Some adenomata were very large and were composed of sheets of cells arranged in broad papillae (Fig. 11). In these the possibility of early neoplastic change could not be excluded, but there was no evidence of local infiltration.

In the mouse with macroscopic cancer histological examination showed complete replacement of the thyroid by a poorly-differentiated adenocarcinoma whose cells were arranged in large papillae in some areas and in irregular acini in others. Colloid was not found in these acini (Fig. 12). The lungs of this mouse contained many metastases throughout their substance and subpleurally ; these were rather better differentiated than the primary tumour, for a few acini contained colloid (Fig. 13). In the other animals no pulmonary deposits were present.

Pituitary enlargement was conspicuous in this group, and in 8 mice large pituitary adenomata were present. One female mouse had a large anaplastic carcinoma of the caecum.

Of the 11 thyroid adenomata 8 occurred in females and 3 in males. The carcinoma, however, occurred in a male mouse.

### DISCUSSION

Both thiouracil administration and a low iodine diet act by interfering with thyroxin synthesis. A deficiency of circulating thyroid hormone causes hyperplasia of those cells of the pituitary responsible for the secretion of thyrotrophic hormone (Russfield, 1955). Frank adenomata of these cells have been produced experimentally in mice given large doses of radioactive iodine (Burt, Landing and Sommers, 1954). The thyrotrophic hormone induces hyperplasia of the thyroid gland.

It is of great interest to determine whether this hyperplasia can proceed to of state of neoplasia, because such tumours would have been induced by an intrinsic alteration of internal environment following a derangement of hormone synthesis rather than by the action of some extrinsic carcinogen.

In the rat such neoplasia has been produced quite easily (Bielschowsky, 1955). Indeed Hall and Bielschowsky (1949) demonstrated that, although 2-acetyl-aminofluorene potentiated the neoplastic effect of thiouracil on rat thyroid during the first year of administration, malignant tumours developed quite as readily without the addition of the carcinogen after 18 months.

In the mouse it is much more difficult to produce thyroid tumours. It is clear that an exuberant pattern of hyperplasia is characteristic, and even the presence of discrete adenomata, valuable indications of progressive cellular proliferation, will not suffice as incontrovertible evidence of neoplastic change. Only definite infiltration of surrounding structures and the presence of distant metastases can be accepted as positive proof of this.

Gorbman (1947) fed mice of 2 strains, A and C57, on a diet containing 0·1 per cent thiouracil. A vast series of changes were noted in the thyroids of these animals, which were killed at periods varying from 7 to 566 days. Of the 22 survivors killed after 500 days, he found pulmonary deposits resembling thyroid tissue in 7, all strain A mice. These little nodules were not accepted as true metastases with neoplastic potentiality, but rather as fragments of hyperplastic thyroid tissue that had acquired an intravascular position in a very active gland, and had been then swept into the venous circulation.

Dalton, Morris and Dubnik (1948) fed 24 strain C3H mice on a diet containing 0·5 per cent thiouracil. After 362 days pulmonary deposits were found in 10 of them. Once again the intravascular position of these foci and their endothelial investment was noted. Repeating the experiment with 32 strain C mice only one instance of pulmonary involvement was encountered, though in these animals nodular hyperplasia of the thyroid was more conspicuous than in strain C3H mice (Dalton, Morris, Striebich and Dubnik, 1950). It was essential to assess the neoplastic nature of this thyroid tissue more conclusively, so Morris and Green (1951) transplanted it into young mice on a diet containing thiouracil. After 3 to 6 months, when the tissue had increased sufficiently in size, it was retransplanted into other mice on a similar diet. After several transplantations there appeared lines of thyroid tissue capable of growth in mice fed on a normal diet. Ultimately some of these tumours metastasised to the lungs and killed the animals. It could now be concluded that autonomy has been attained, in that growth could occur without any additional requirement of thyrotrophic hormone.

In the present experiment a correlation between the degree of induced thyroxin deficiency and the gamut of hyperplastic response has been made. The administration of methylthiouracil alone produced profound hyperplasia of a characteristically papillary type. When it was combined with a low iodine diet, focal papillary adenomata were encountered in almost half the thyroids examined. The gradual transformation from diffuse acinar hyperplasia with papillary ingrowths to fully established papillary hyperplasia is followed in turn by the appearance of nodules of extreme hyperplasia. It is in these areas that focal adenomata arise.

The difficulty in inducing malignant change in such glands is again emphasised, though in one case there was clear-cut and decisive cancerous transformation. Had the investigation been terminated later, it is quite possible that malignancy might have been encountered more frequently. To this purpose another group of mice is being treated similarly at present and in these the extreme thyroxin deficiency will be allowed to act for a greater period of time.

### SUMMARY

One hundred C57 mice were divided into 3 groups :

(*a*) A control group of 25 mice fed on a stock diet and tap water.

(*b*) A group of 50 mice fed on a stock diet and distilled drinking water containing 0·05 per cent methylthiouracil.

(*c*) A group of 25 mice fed on a low iodine diet and distilled drinking water containing 0·05 per cent methylthiouracil.

After 480 days no significant changes were found in the thyroid glands of the control group.

In the second group extreme hyperplasia of a typically papillary type was encountered, but there was no evidence of neoplasia.

In the third group focal papillary adenomata were found in 11 of the 25 mice, but in none was there any tendency to local infiltration or distant metastasis. One mouse, however, did succumb to an adenocarcinoma of the thyroid that metastasised to the lungs.

The extreme difficulty in producing thyroid neoplasms in mice even under the most intense stimulation is demonstrated once again.

We are grateful to Professor G. J. Cunningham for his interest and assistance ; to Miss June Hunter, of the Medical School, University of Otago, New Zealand, for help in planning the experiment ; to Dr. J. Craigie, of the Imperial Cancer

---

### EXPLANATION OF PLATES

FIG. 1.—Normal thyroid gland in one of the control group. H. and E. ×65.

FIG. 2.—Pronounced acinar hyperplasia with papillary invaginations into some acini. H. and E. ×65.

FIG. 3.—A later stage in the evolution of papillary hyperplasia from acinar hyperplasia. H. and E. ×65.

FIG. 4.—The pattern of fully-developed papillary hyperplasia. H. and E. ×65.

FIG. 5.—A circumscribed nodule of papillary hyperplasia resembling an adenoma. The cellular pattern is quite uniform. H. and E. ×65.

FIG. 6.—The regular cellular arrangement in papillary hyperplasia. H. and E. ×200.

FIG. 7.—More pronounced hyperplastic pattern with some large cells having giant nuclei. H. and E. ×200.

FIG. 8.—Solid cell hyperplasia. H. and E. ×70.

FIG. 9.—A circumscribed papillary adenoma. The arrangement and morphology of the cells is quite different from that of the surrounding tissue. H. and E. ×70.

FIG. 10.—The distinctive cellular structure of a papillary adenoma. H. and E. ×200.

FIG. 11.—A particularly exuberant papillary adenoma. H. and E. ×70.

FIG. 12.—A rather poorly differentiated adenocarcinoma showing a papillary pattern in some areas and an acinar one elsewhere. H. and E. ×70.

FIG. 13.—A pulmonary metastasis. It has a much better preserved acinar pattern than the primary tumour. H. and E. ×70.

1

2

3

4

5

6

7

8

9

10

11

12

13

Research Fund, for advice about the low iodine diet ; and to Mr. A. L. E. Barron for the photomicrographs.

### REFERENCES

ASTWOOD, E. B., SULLIVAN, J., BISSELL, A. AND TYSLOWITZ, R.—(1943) *Endocrinology*, **32**, 210.

AXELRAD, A. A. AND LEBLOND, C. P.—(1952) *Canad. med. Ass. J.*, **67**, 675.—(1955) *Cancer*, **8**, 339.

BIELSCHOWSKY, F.—(1955) *Brit. J. Cancer*, **9**, 80.

BURT, A. S., LANDING, B. H. AND SOMMERS, S. C.—(1954) *Cancer Res.*, **14**, 497.

DALTON, A. J., MORRIS, H. P. AND DUBNIK, C. S.—(1948) *J. nat. Cancer Inst.*, **9**, 201.

*Idem*, MORRIS, H. P., STRIEBICH, M. J. AND DUBNIK, C. S.—(1950) *Ibid.*, **11**, 391.

GORBMAN, A.—(1947) *Cancer Res.*, **7**, 746.

HALL, W. H. AND BIELSCHOWSKY, F.—(1949) *Brit. J. Cancer*, **3**, 534.

MORRIS, H. P. AND GREEN, C. D.—(1951) *Science*, **114**, 44.

REMINGTON, R. E.—(1937) *J. Nutr.*, **13**, 223.

RUSSFIELD, A. B.—(1955) *J. clin. Endocrin.*, **15**, 1393.